# CARE TRANSITIONS FROM HOSPITAL TO HOME: PERSPECTIVES FROM OLDER ADULTS, CAREGIVERS, AND HEALTHCARE PROFESSIONALS

**DOI:** 10.1093/geroni/igae098.3821

**Published:** 2024-12-31

**Authors:** Taghreed Alotaibi, Judith Sixsmith, roseanne cetnarskyj, mei Fang

**Affiliations:** Dundee University, Dundee, Scotland, United Kingdom; University of Dundee, Dundee, Scotland, United Kingdom; Dundee University, Dundee, Scotland, United Kingdom; SIMON FRASER UNIVERSITY, Simon Fraser University, British Columbia, Canada

## Abstract

**Background:**

Saudi Arabia switched from hospital-focused to home-based care for older persons with long-term care needs due to a 25% increase in acute hospital bed occupancy for non-acute older adults. Healthcare professionals, older adults, and families are affected by the change in the care transition. Improvement in health outcomes depends on understanding all three groups.

**Aim:**

This study aims to investigate the hospital-to-home transition of older adults with chronic conditions. The objective is to identify factors that facilitate or hinder the transition, with the aim of improving care during this critical phase.

**Methods:**

The research employed a descriptive case study analysed with thematic analysis and an institutional ethnography. Pre- and post-discharge data were obtained from older adults, carers, and healthcare workers at King Saud Medical City using semi-structured interviews, focus groups, participatory observation, and documentary analysis.

**Findings:**

The findings indicate that culture, family, and national healthcare policies play a crucial role. Older adults and their caregivers encounter obstacles during the transition process, including gender segregation causing difficulties with care coordination. The lack of standardised post-discharge follow-up and fragmented care provision further contribute to these hurdles.

**Conclusion:**

The research recommends creating a unified administration system as a necessary foundation for solving the largest problems in the transition. This may help Saudi Arabia’s care policymakers and planners create a family-centred healthcare system. Future research should focus on targeted strategies to improve the care transition experiences of older adults and their caregivers.

